# Supplementary material for: SpPKE1, a Multiple Stress-Responsive Gene Confers Salt Tolerance in Tomato and Tobacco
Source: Int J Mol Sci. 2019 May 20;20(10):2478. doi: 10.3390/ijms20102478 (PMC6566969; doi:10.3390/ijms20102478)
Supplement: Supplementary file 1 [file ijms-20-02478-s001.zip › Supplementary Material.pdf]

Supplementary Material:

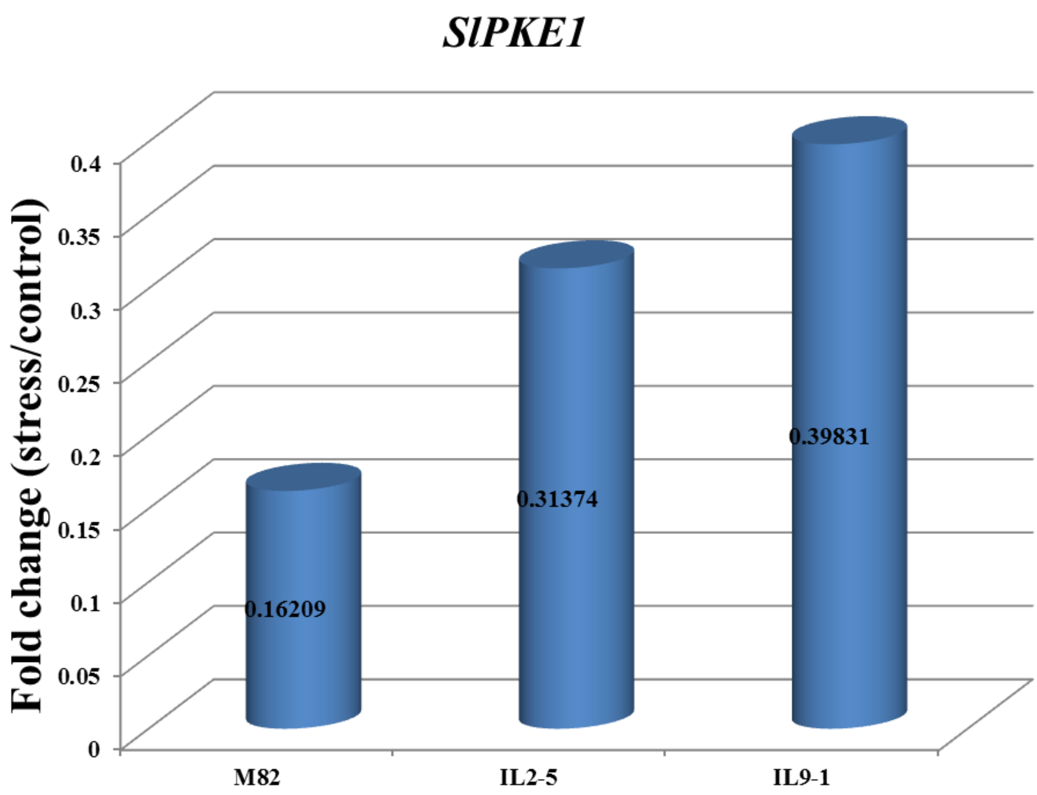

Figure S1 *SlPKE1* was responsive to drought stress in three genotypes (M82, IL2-5 and IL9-1) in microarray data [16]. Genes (FDR <0.05 and fold>=2) were identified as differentially expressed genes.

Table S1. Sequence composition of SpPKE1 and SlPKE1.

SpPKE1 / Amino acid	Abbreviation	Number	Frequency (%)	SlPKE1 / Amino acid	Abbreviation	Number	Frequency (%)
Pro	P	59	18.5	Pro	P	61	18.7
Lys	K	52	16.3	Lys	K	56	17.2
Glu	E	36	11.3	Glu	E	40	12.3
Val	V	27	8.5	Val	V	30	9.2
Gly	G	20	6.3	Gly	G	19	5.8
Tyr	Y	15	4.7	Tyr	Y	15	4.6
Ile	I	15	4.7	Ile	I	15	4.6
Cys	C	15	4.7	Cys	C	15	4.6
Gln	Q	13	4.1	Gln	Q	14	4.3
Ser	S	12	3.8	Ser	S	11	3.4
Leu	L	9	2.8	Leu	L	8	2.5
Asp	D	8	2.5	Asp	D	8	2.5
Ala	A	7	2.2	Ala	A	7	2.1
Met	M	7	2.2	Met	M	6	1.8
Thr	T	6	1.9	Thr	T	6	1.8
Asn	N	6	1.9	Asn	N	6	1.8
His	H	4	1.3	Phe	F	3	0.9
Arg	R	4	1.3	Arg	R	3	0.9
Phe	F	3	0.9	His	H	2	0.6
Trp	W	1	0.3	Trp	W	1	0.3

Table S2 Primer sequences used for vector construction and qRT-PCR analysis.

Primer		Sequence (5'-3')
PKE-OE	Overexpression	Forward (Fw): TCCCTTCTCAAGTTTACCCAAA<br>Reverse (Rv): TTGGTGAAAAGGCCCAATTA
35S	Overexpression	Fw: TTCGCAAGACCCTTCCTCTA
PKE1(Q)	qRT-PCR	Fw: GAAGCACCTAAACCAGCAC<br>Rv: TAGCCTTCGTAACATTGCC
β-Actin	qRT-PCR	Fw: GTCCTCTTCCAGCCATCCAT<br>Rv: ACCACTGAGCACAATGTTACCG
PKE1-SL	Subcellular localization	Fw: GGTACC TCTTAGTTCTCAACTATGGGTG<br>Rv: GGATCC CATAATTGAGCATCCAGATG
